# Genome-wide analysis reveals differential selection involved with copy number variation in diverse Chinese Cattle

**DOI:** 10.1038/s41598-017-14768-0

**Published:** 2017-10-30

**Authors:** Liu Yang, Lingyang Xu, Bo Zhu, Hong Niu, Wengang Zhang, Jian Miao, Xinping Shi, Ming Zhang, Yan Chen, Lupei Zhang, Xue Gao, Huijiang Gao, Li Li, George E. Liu, Junya Li

**Affiliations:** 1 Laboratory of Molecular Biology and Bovine Breeding, Institute of Animal Sciences, Chinese Academy of Agricultural Sciences, Beijing, 100193 China; 2 Farm Animal Genetic Resources Exploration and Innovation Key Laboratory of Sichuan Province, Sichuan Agricultural University, Chengdu, Sichuan, 611130 China; 3 College of Animal Sciences, Fujian Agriculture and Forestry University, Fuzhou, Fujian, 350002 China; 4 College of Animal Science and Technology, Agricultural University of Hebei, Baoding, Hebei, 071001 China; 5 Animal Genomics and Improvement Laboratory, Agricultural Research Service, USDA, Beltsville, Maryland, 20705 USA

## Abstract

Copy number variations (CNVs) are defined as deletions, insertions, and duplications between two individuals of a species. To investigate the diversity and population-genetic properties of CNVs and their diverse selection patterns, we performed a genome-wide CNV analysis using high density SNP array in Chinese native cattle. In this study, we detected a total of 13,225 CNV events and 3,356 CNV regions (CNVRs), overlapping with 1,522 annotated genes. Among them, approximately 71.43 Mb of novel CNVRs were detected in the Chinese cattle population for the first time, representing the unique genomic resources in cattle. A new *V*_*i*_ statistic was proposed to estimate the region-specific divergence in CNVR for each group based on unbiased estimates of pairwise *V*_*ST*_. We obtained 12 and 62 candidate CNVRs at the top 1% and top 5% of genome-wide *V*_*i*_ value thresholds for each of four groups (North, Northwest, Southwest and South). Moreover, we identified many lineage-differentiated CNV genes across four groups, which were associated with several important molecular functions and biological processes, including metabolic process, response to stimulus, immune system, and others. Our findings provide some insights into understanding lineage-differentiated CNVs under divergent selection in the Chinese native cattle.

## Introduction

Copy number variations (CNVs) are defined as deletions, insertions, and duplications ranging from 50 base pairs (bp) to 5 million base pairs (Mbp) of genomic sequence between two individuals of a species^1–4^. Previous studies suggest CNV have potentially larger effects than other variation such as SNP, including changing gene structure and dosage, altering gene regulation and exposing recessive alleles^5–7^. CNV discovery studies have been extensively reported in human^8^, primates^9^, mouse^10–12^, zebrafish^13^, dog^14–16^, and livestock, including chicken^17,18^, pig^19,20^, sheep^21–23^, goat^24^ and cattle^25–29^.

In recent years, many studies have revealed genomic diversity could be generated by the differential selection of CNVs in specific environments for adaptations^30–33^. In human, positive selection for a higher *AMY1* copy number enables better digestion of starchy foods^34^. A change in *CCL3L1* copy number is associated with markedly enhanced HIV/acquired immunodeficiency syndrome (AIDS) susceptibility^35^. The human *UGT2B17* gene shows significant copy-number diversity, and displays region-specific differences for metabolism in multiple populations^36^. Also, olfactory receptor (OR) genes with variable copy numbers among most mammalian species were found associate with population-specific differences in smell^37^. CNVs are specifically enriched among evolutionary “young” ORs, implying that CNVs may play an essential role in the origin of a gene or the emergence of new OR gene clusters^38^.

Domesticated cattle are one of the most economically important farm animals. The exploration of genetic diversity, conservation, selection and evolution of genomic variants in cattle have attracted much attention in past decades^39^. Specifically, investigations of population genetic properties and selection patterns involved with CNVs have gradually become an emerging research topic for farm animals. For instance, Xu *et al*. have investigated the population-genetic properties of differentiated CNVs using high density SNP array among European taurine, African taurine, and indicine groups, and provided a list of lineage-differentiated CNVs, which were involved in traits related to parasite resistance, immunity response, body size, fertility, and milk production^40^. Bickhart *et al*. further explored the diversity and population genetics of both taurine and indicine cattle based on CNV using next generation sequencing and showed hundreds of copy number variable genes were breed-specific^31^. Although a few studies have been carried out to investigate CNV in Chinese cattle^41–43^, genome-wide CNV landscapes and its population-genetic properties in Chinese cattle adapted for local specific environments are largely unknown.

In this study, we performed a genome-wide CNV analysis using high density SNP array in diverse Chinese cattle populations. The objectives of this study were to 1) Generate a comprehensive CNV landscape in Chinese cattle populations; 2) Investigate and compare the diversity and population-genetic properties of CNVs; 3) Explore the diverse selection patterns involved with CNV genes for local adaptation in Chinese native cattle.

## Results

### CNVs identification

**Figure 1:**
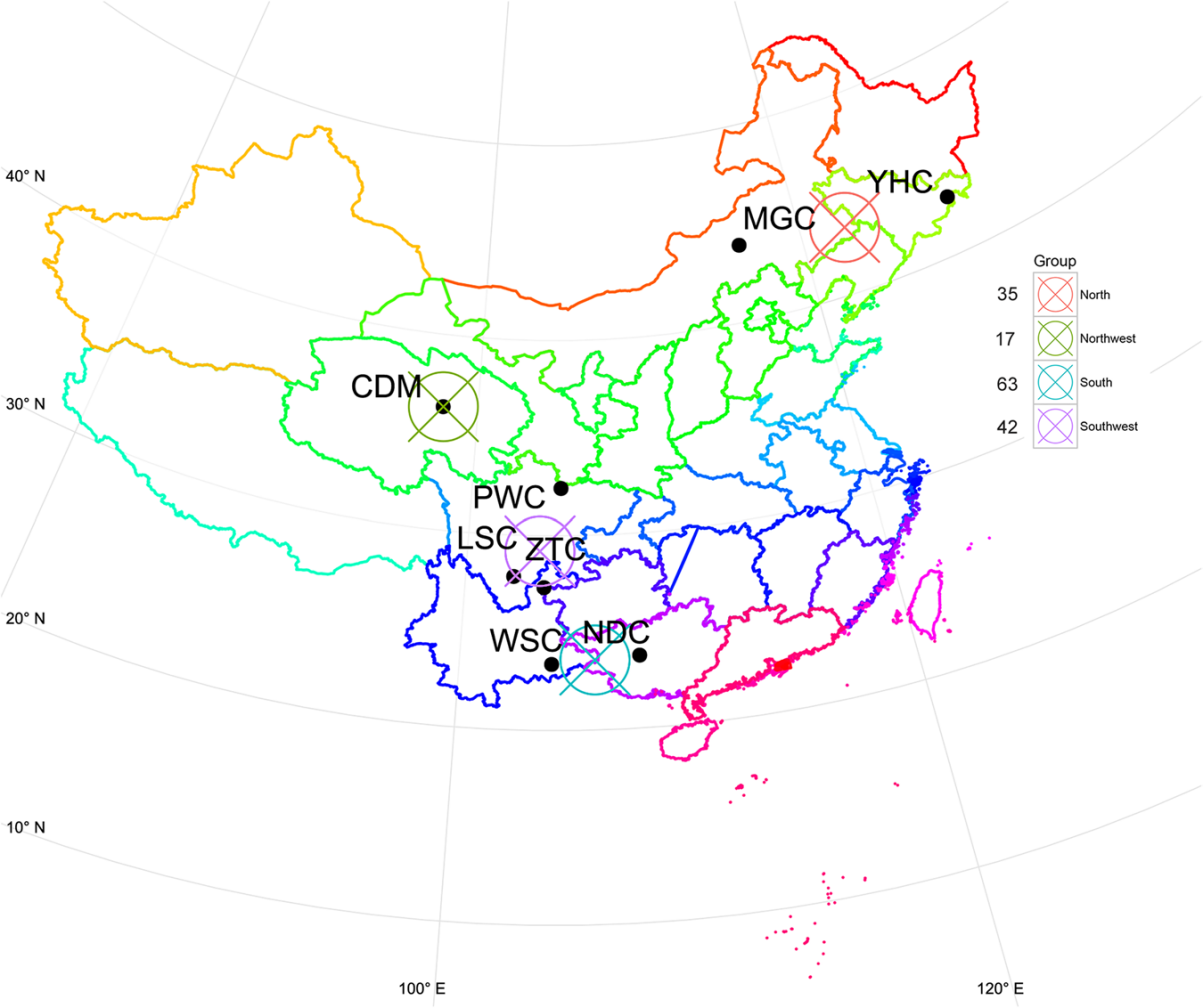
Geographic distribution of four groups from eight populations in China. YHC, MGC, CDM, PWC, LSC, ZTC, WSC and NDC are abbreviations for Yanhuang, Monggu, Caidamu, Pingwu, Liangshan, Zhaotong, Wenshan and Nandan, respectively. The numbers of individual sample for each group were shown beside figure legend. The distribution of cattle was summarized and visualized with R packages maps, mapproj, maptools and ggplot 2.

**Figure 2:**
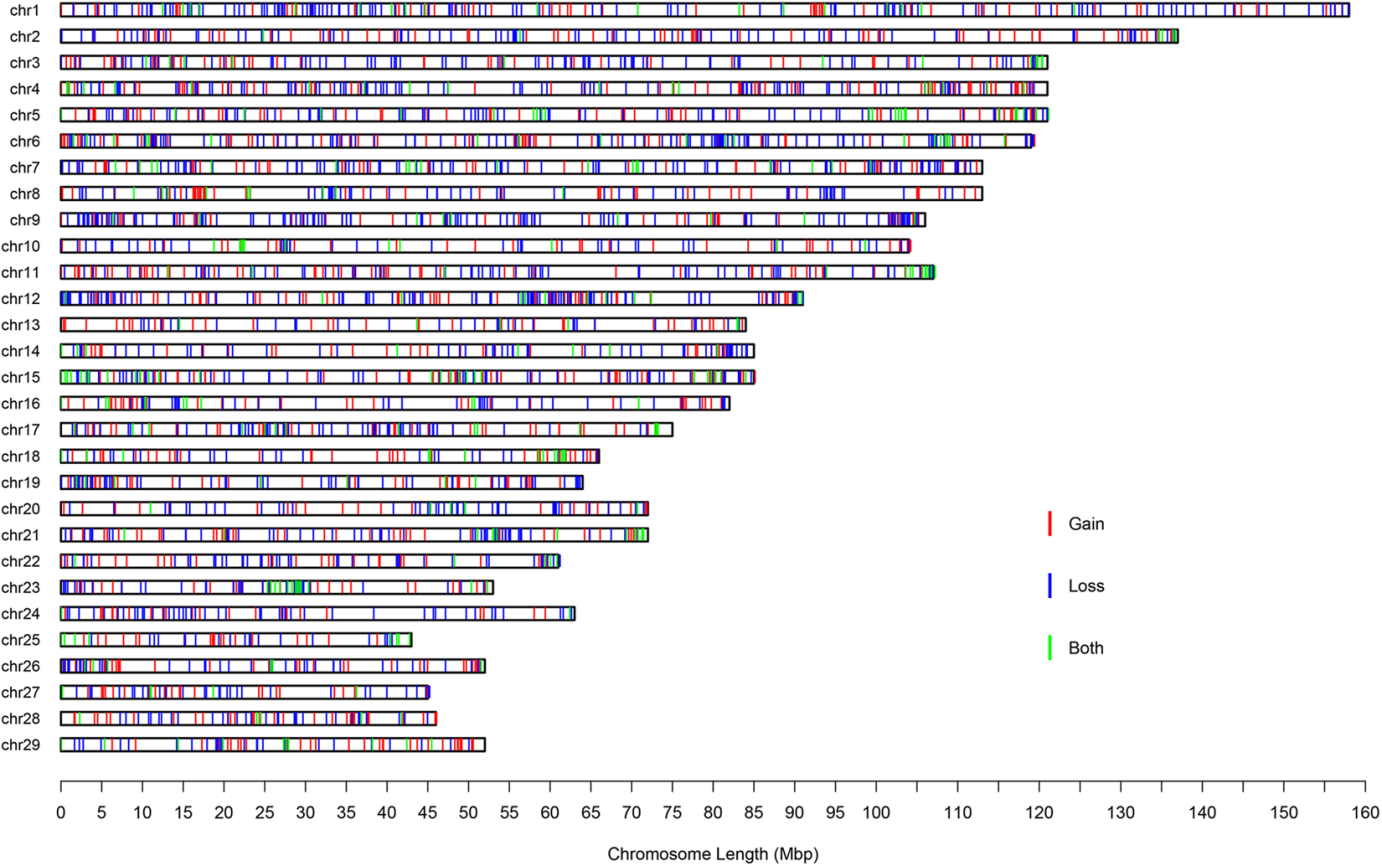
Genomic distribution and status of detected CNVRs in 157 cattle. Red, green, and blue lines represent the predicted statuses of gain, loss, and both, respectively. A total of 13,325 CNVs were merged into 3,356 copy number variant regions (CNVRs), covering 148.0 Mb (average of 44.1 kb) of polymorphic sequence. Among them, 2,124 “unique” (only present in one individual), 1,278 “gain” (account for 38.1%), 1,748 “loss” (52.1%) and 330 “both” (9.8%) were identified in our analysis.

We performed a genome-wide CNV analysis using the Illumina Bovine HD SNP array in 188 individuals from eight Chinese cattle populations (Fig. 1). After filtering by call rate and genetic relationship, a total of 167 individuals remained. Autosomal CNVs were identified following standard analysis procedures using PennCNV based on the taurine reference assembly (UMD3.1). After CNV detection, low-quality samples were filtered out. A total of 157 individuals were finally used for downstream analyses. Our study detected a total of 13,325 CNVs, representing an average length of 61.2 kb across all individuals (Table 1). These CNVs were merged into 3,356 copy number variant regions (CNVRs), covering 148.0 Mb (average of 44.1 kb) of polymorphic sequence, and corresponding to 5.81% of autosomal genome (148/2,545.9) and 5.07% (148/2,918.0) of the whole genome (Fig. 2 and Supplementary File 1: Table S1). Among them, 2,124 “unique” (only present in one individual), 1,278 “gain” (account for 38.1%), 1,748 “loss” (52.1%) and 330 “both” (9.8%) were identified in our analysis. Notably, we found the count of loss event was approximately 1.4-fold more than gain events, and 5.3-fold more than both events, however, the average length of “both” CNVRs (176.1 kb) were larger than “loss” (32.3 kb) and “gain” (26.2 kb).

**Table 1:** CNV events and CNVR detected in four groups (North, Northwest, Southwest and South groups).

Group	CNV				Sample Size	CNVR					
	CNV	Gain	Loss	Length		CNVR	Unique	Gain	Loss	Both	Length
Total	13,325 (84.87)	4,534	8,791	814,865,357 (61,153)	157	3,356	2,124	1,278	1,748	330	148,099,076 (44,130)
North	2,600 (74.29)	940	1,660	184,528,642 (70,973)	35	969	658	329	557	83	60,838,371 (62,785)
Northwest	1,254 (73.76)	511	743	72,509,715 (57,823)	17	544	373	258	240	46	27,810,707 (51,123)
Southwest	5,472 (86.86)	2,100	3,372	308,854,760 (56,443)	63	1,880	1,259	841	886	153	84,525,087 (44,960)
South	3,999 (95.21)	983	3,016	248,972,240 (62,259)	42	1,401	943	364	934	103	62,497,649 (44,609)

Numbers in parentheses represent per sample averages except in the case of the “Length” column which is the average length. The number of CNVR (Gain, Loss and Both) represent nonredundent CNVR counts.

**Figure 3:**
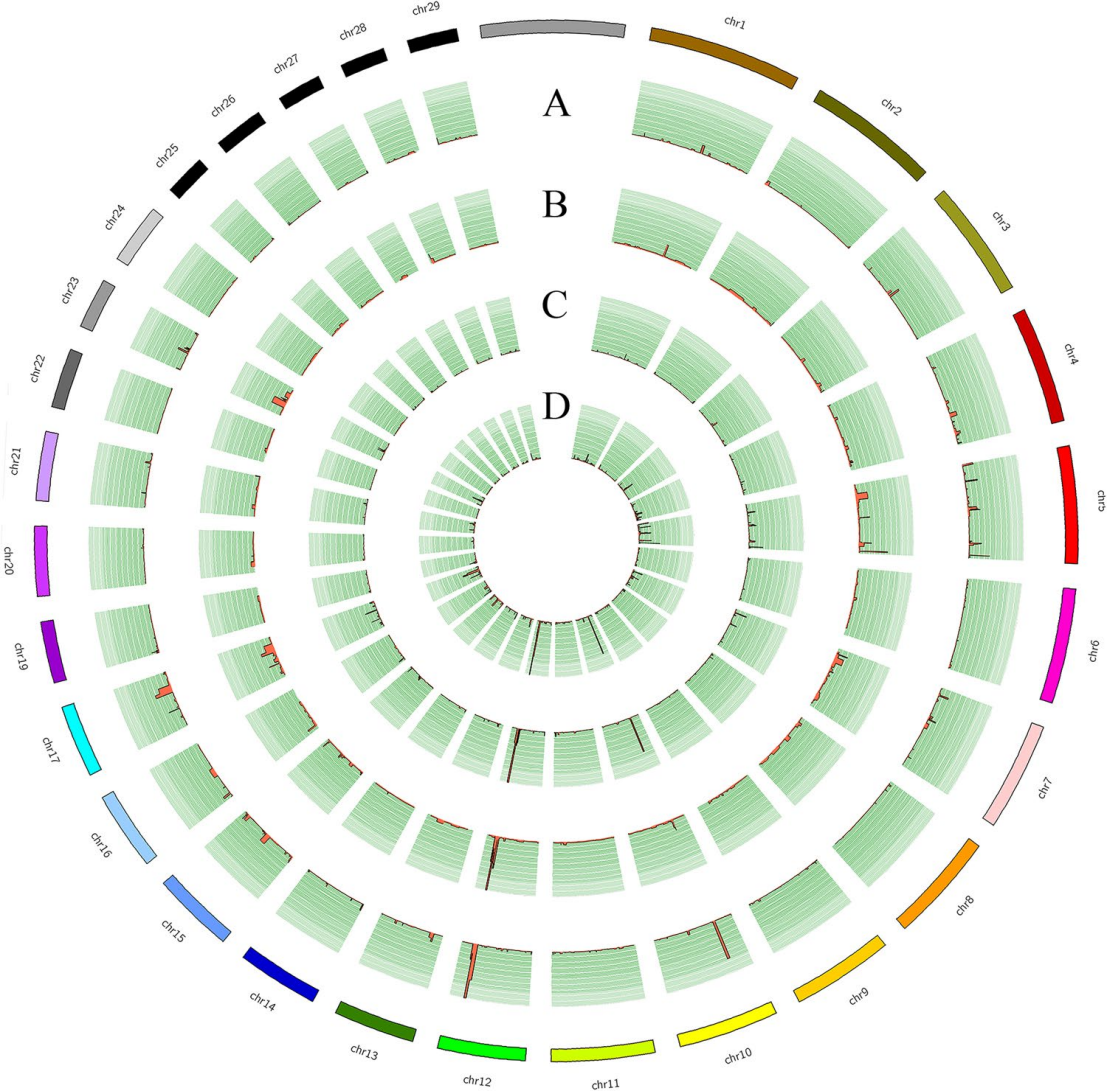
Circos plot illustrating CNV regions in 4 groups of cattle. The circles from outside to inside represent frequency of CNV event in each CNVR from North (**A**), Northwest (**B**), South (**C**) and Southwest (**D**) group respectively.

Next, we divided the 157 individuals from eight populations into 4 groups which consisted of North (n = 35), Northwest (n = 17), Southwest (n = 63) and South (n = 42) based on the Multidimensional scaling (MDS) analysis results based on SNPs (Figure S1). The CNV events among individuals within each group were merged into group-specific CNVRs. We observed 969, 544, 1880 and 1401 CNVRs in North, Northwest, Southwest and South groups with 62.8, 51.1, 45.0 and 44.6 kb of average length, respectively (Supplementary File 1: Table S1). To display the distribution of CNVR across genome, we filtered away CNVRs with only one CNV event, these single CNV events are more likely to be false-positive CNVs which are not real, and finally 1,232 CNVRs remained. Based on the 1,232 CNVRs, we generated a Circos plot to visualize CNVR landscapes across groups (Fig. 3).

### CNVR annotation and enrichment analysis

To explore the potential function involved with CNV genes in Chinese cattle, we performed functional annotation and GO enrichment analysis of CNV genes using PANTHER. A total of 1,400 unique genes were found overlapped with 3,356 CNVR, and these identified genes were further used for PANTHER enrichment analysis. We found the identified CNV regions were mainly enriched in fatty acid beta-oxidation, catabolic process, catabolic process, transport, localization, system process, neurological system process and G-protein coupled receptor signaling pathway sensory perception (Supplementary File 2: Table S2). Simultaneously, we carried out the gene feature analysis using the detected CNV genes, and we observed a total of 891 CNVRs and 679 CNVRs overlapping with 1,320 CDSs (coding sequences) and 1,189 exons, respectively.

### Selection signatures of CNVs

**Figure 4:**
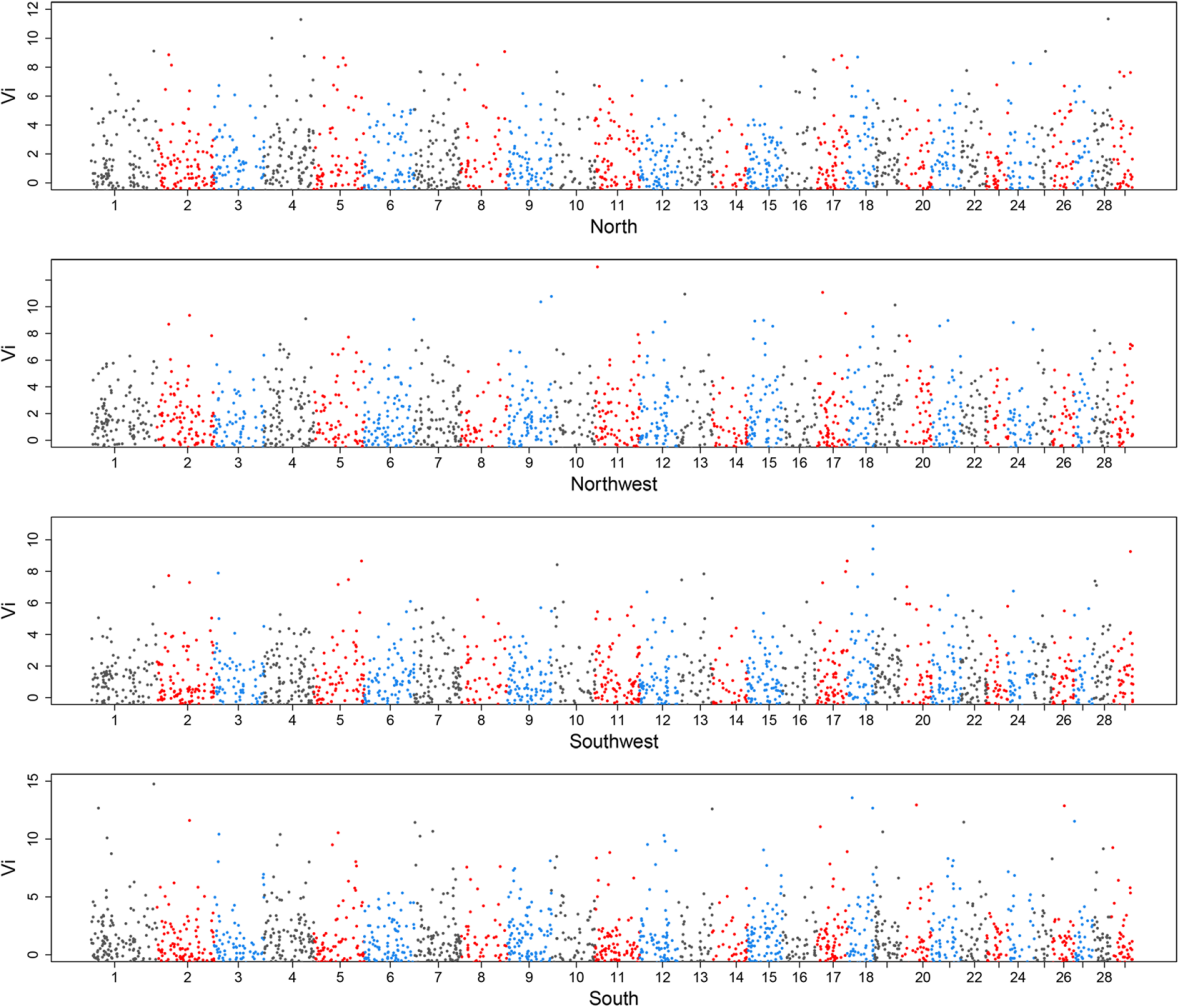
Genome-wide distribution of *V*_*i*_ values among North, Northwest, Southwest and South groups. The *V*_*i*_ value was defined as a function of unbiased estimates of all pairwise *V*_*ST*_ between one group and the remaining groups within a population, and *V*_*i*_ statistic was suitable for detecting selection specific involved with CNVs to a particular group. The distribution of *V*_*i*_ for each CNVR across all auto chromosomes is shown for each group. Alternating color indicates *V*_*i*_ values from adjacent chromosomes.

To investigate the group-specific selection involved with CNV events, *V*_*i*,_ a new statistic modified from *d*_*i*_, was proposed to characterize group differentiation of CNVs. The *V*_*i*_ value was defined as a function of unbiased estimates of all pairwise *V*_*ST*_ between one group and the remaining groups, and *V*_*i*_ statistic was suitable for detecting selection specific involved with CNVs to a particular group. In this study, we performed a genome-wide scan for differentiation analysis using *V*_*i*_ in four groups (North, Northwest, Southwest and South). We then generated Manhattan plots of *V*_*i*_ distribution for four groups as shown in Fig. 4 (Summary statistics were presented in Supplementary File 3: Table S3). After filtering away CNVRs with only one CNV event, 1,232 CNVRs remained. For each group, we defined candidate regions involved with selection using two thresholds: top 1% or 5% of CNVRs with highest *V*_*i*_ values in the empirical distribution. In this study, we finally obtained 12 and 62 candidate CNVRs at the top 1% and top 5% for each group, and threshold of *V*_*i*_ value for top 1% were 6.70, 7.19, 6.07 and 9.25 in North, Northwest, Southwest and South group, while the threshold for top 5% were 3.80, 4.62, 3.32 and 4.95, respectively.

**Figure 5:**
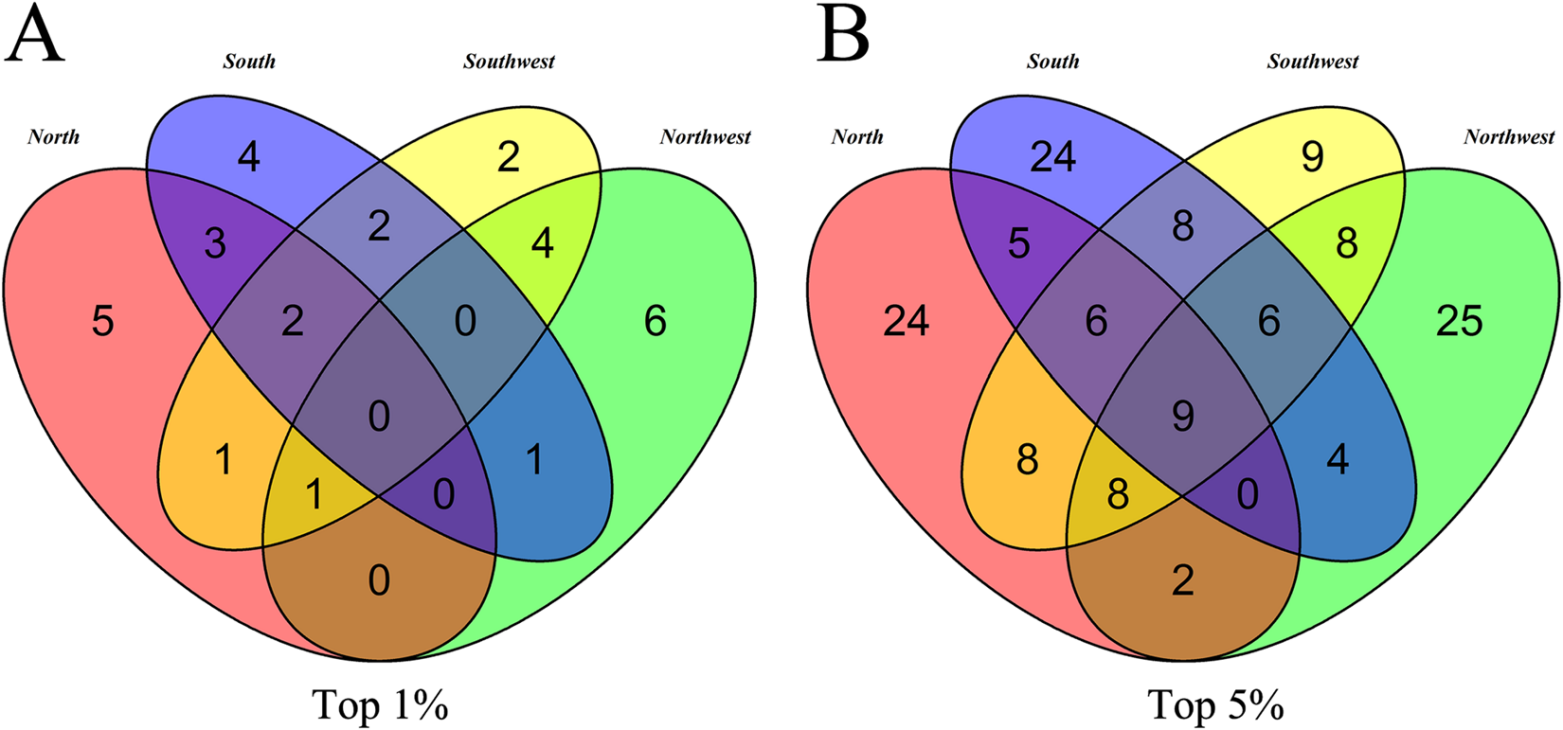
Discovery of shared and group-specific CNVRs among 4 groups. The Venn diagram shows the number of overlapping CNVRs in 4 groups including North, Northwest, Southwest and South groups. The top 1% (n = 12) and top 5% (n = 60) of CNVRs among four groups were displayed in left and right, respectively.

**Figure 6:**
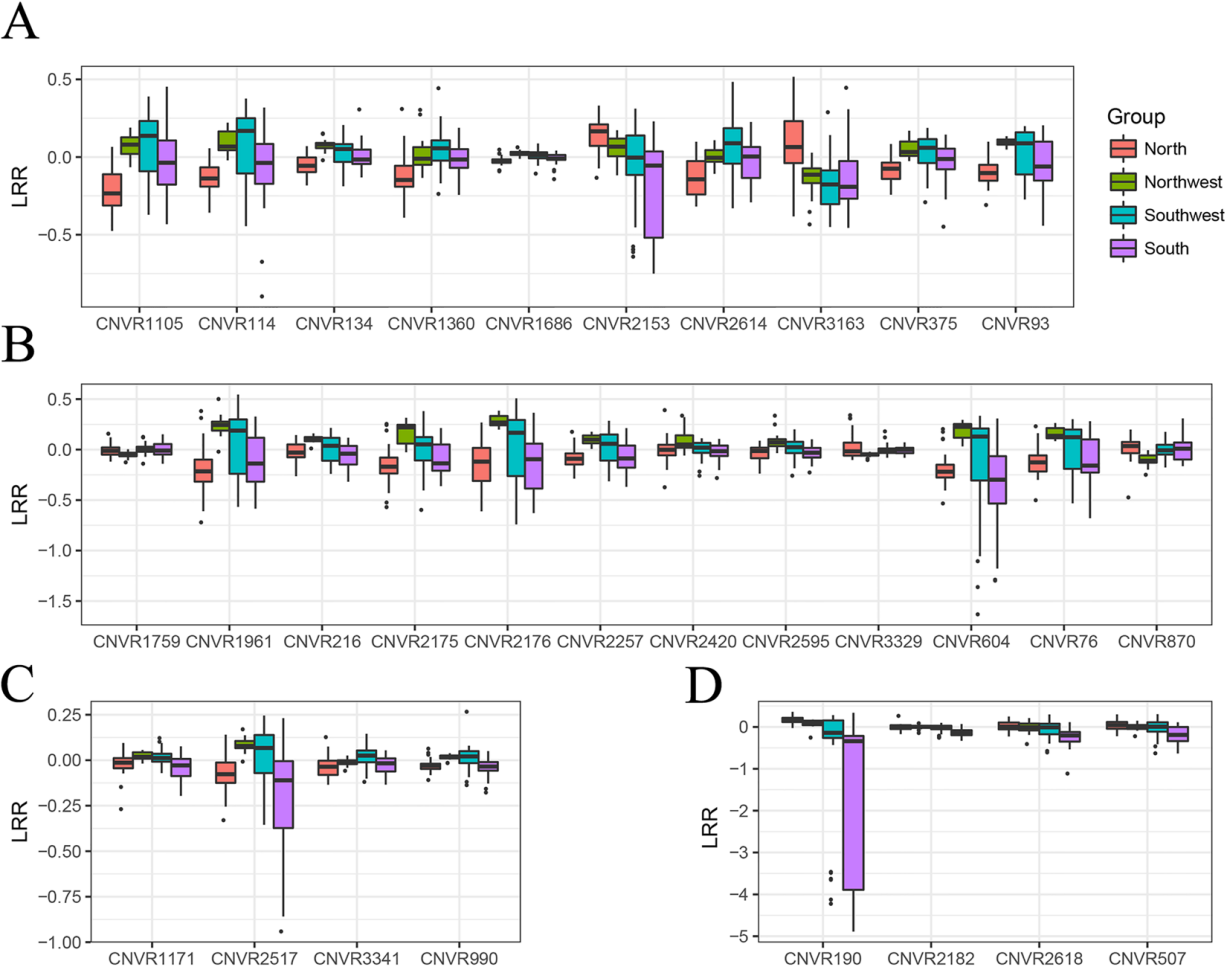
Box-plot of average LRR values for group-specific CNVRs across four groups. (**A**) North-specific group, (**B**) Northwest-specific group, (**C**) Southwest-specific group, (**D**) South-specific group.

To identify shared and group-specific CNVRs under potential selection, we next generated the Venn diagram based on these CNVRs (Fig. 5). At top 1%, 5, 4, 2 and 6 of group-specific CNVRs were found in North, South, Northwest and Southwest groups, respectively, while no shared CNVR was found. Next, we relaxed the threshold from top 1% to top 5%, we observed 9 CNVRs were shared by all 4 groups, 82 CNVRs were identified as group-specific CNVRs, while 24 CNVRs were detected in North, 24 in South, 25 in Northwest and 9 in Southwest group, respectively. To further estimate the divergence of CNVs across groups for the identified candidate CNVRs, we extracted the LRR values for each shared and unique CNVRs in four groups. Notably, we observed clear differences based on average LRR for each group-specific CNVR and the Box-plot of the distributions of average LRR in the identified CNVRs across diverse cattle groups were shown in Fig. 6.

### Genes in shared and group-specific CNVRs

To investigate lineage-differentiated CNV genes in Chinese cattle, we also conducted CNVR-based population differentiation analysis, and identified a set of potential CNV genes under divergent selection (Table 2). In the shared CNVRs, we detected 4 genes embedded with 3 CNVRs, among them, CNVR208 and CNVR2872 on BTA1 and BTA22 with length of 14.6 and 15.0 kb, overlapping gene *CBR1* and *TKT*, respectively, and CNVR2451 on BTA17 with length of 71.4 kb spanning two genes *LOC100297192* and *IGLL1*.

**Table 2:** Summary statistics of shared and group-specific CNVRs, including the position of CNVR, estimated *V*_*i*_ value for each group and corresponding CNV genes.

Group	CNVR	Chr	Start	End	*V*_*i*_ -North	*V*_*i*_ -Northwest	*V*_*i*_ -Southwest	*V*_*i*_ -South	Candidate Genes
Shared	208	1	150050992	150065547	9.11	5.90	7.03	14.76	CBR1
	2451	17	72870233	72941663	7.96	6.36	8.65	8.91	IGLL1,LOC100297192
	2872	22	48265166	48280157	4.22	4.78	5.07	5.02	TKT
North	93	1	44683130	44743553	4.89	4.02	1.67	0.74	EFHB
	114	1	58435550	58449521	4.97	2.37	2.20	0.44	BOC
	134	1	83760081	83775860	4.99	3.34	1.77	1.00	ABCC5
	375	3	1667959	1704027	5.24	1.80	2.44	0.73	DUSP27
	1105	7	16054798	16073843	7.66	1.65	2.65	1.12	FDX1L,ICAM5,RAVER1
	1360	8	105256615	105273649	4.44	−0.95	3.10	0.82	COL27A1
	1686	11	38648841	38851335	4.32	2.85	3.07	1.60	CCDC85A
	2153	15	17038087	17074274	4.00	0.51	1.85	4.88	GUCY1A2
	2614	19	54639709	54648592	4.42	−0.06	2.58	0.52	TMC6
	3163	27	5444267	5973102	5.60	−0.88	1.76	0.01	DEFB,DEFB1,DEFB5,EBD
Northwest	76	1	36710870	36729816	2.72	5.52	1.56	1.51	EPHA3
	216	1	157196044	157225181	0.53	5.44	1.17	2.31	SATB1
	604	4	46975403	46982930	2.77	6.81	0.97	2.12	ATXN7L1
	870	5	119430708	119531892	−0.77	5.15	2.13	−0.95	FAM19A5
	1759	11	104195124	104384812	−1.18	4.90	2.74	−1.26	ABO,MED22,RPL7A,SURF4,SURF6
	1961	13	16187783	16193492	3.24	5.43	2.73	2.86	KIN
	2175	15	42588302	42594246	3.08	6.40	2.71	2.71	MRVI1
	2176	15	42639417	42644258	2.65	7.25	3.14	3.29	MRVI1
	2257	16	933282	950232	2.28	4.89	1.88	1.96	FMOD
	2420	17	43069432	43085729	−1.14	5.01	2.17	1.08	GLRB
	2595	19	41945417	41951980	0.89	4.86	2.42	2.41	LOC618938
	3329	29	37219773	37404822	−0.72	4.88	2.00	0.41	MIR584-7
Southwest	990	6	71630923	72077236	2.09	2.75	3.40	4.49	KIT
	1171	7	64638445	64705407	0.17	0.78	3.78	4.51	FAT2,SLC36A1
	2517	18	60990841	61031513	3.12	2.86	4.19	4.62	ZNF331
	3341	29	45491233	45608617	0.57	−0.68	4.11	0.56	LRFN4,MIR2408,PC,RCE1
South	190	1	130623810	130626113	3.11	1.21	2.26	5.14	KPNA6
	507	3	118059058	118090512	1.51	−0.50	1.52	6.66	ESPNL,SCLY
	2182	15	46571384	47009702	1.33	0.08	2.31	7.71	LOC100125776,LOC506989,LOC530354,MRPL17
	2618	19	56993243	57020892	1.97	−0.68	1.35	6.63	ATP5H

Candidate genes were defined as the genes overlapping with CNVR.

In this study, a total of 30 group-specific CNVRs were overlapped with 46 annotated genes for 4 groups (Table 2). In North group, we obtained 10 CNVRs covering 15 genes, which were mainly associated with metabolic process (*GUCY1A2*, *RAVER1*, *EBD*, *DEFB1* and *DEFB5*) and response to stimulus (*GUCY1A2*, *EBD*, *DEFB1*, *DEFB5* and *ABCC5*). Moreover, gene *TMC6* was related to immune system in CNVR2614 (*V*_*i*_ = 4.42), and *COL27A1* within CNVR1360 (*V*_*i*_ = 4.44) and *BOC* gene within CNVR114 (*V*_*i*_ = 4.97) were identified as North-specific candidates. In addition, we detected several genes including calcium ion binding (*EFHB*), phosphatase activity (*DUSP27*) and GTP binding and phosphorus-oxygen lyase activity (*GUCY1A2*). In Northwest group, a total of 15 genes were obtained in 12 CNVRs, among them, most genes were related to metabolic process (*KIN*, *RPL7A*, *SATB1*, *ABO* and *FMOD*), cellular process (*KIN*, *GLRB*, *SATB1* and *FMOD*), response to stimulus (*KIN* and *GLRB*) and developmental process (*EPHA3* and *FMOD*). Gene *MRVI1* was simultaneously overlapped with CNVR2175 and CNVR2176 with *V*_*i*_ = 6.40 and *V*_*i*_ = 7.25. For Southwest group, we detected 8 genes in 4 group-specific CNVRs. The genes *FAT2* and *SLC36A1* overlapped with CNVR1171 (*V*_*i*_ = 3.78), and CNVR3341 overlapped with three genes including *LRFN4*, *MIR2408*, *PC* and *RCE1*. We also obtained CNVR990 and CNVR2517, which overlapped with gene *KIT* and *ZNF331*, respectively. *KIT* was identified in CNVR990 (*V*_*i*_ = 3.40) in Southwest group, which was also shown to have high *V*_*i*_ value (4.49) in South group. We found 4 group-specific CNVRs with 8 genes in South group, most of these genes involved with cellular process, biological regulation and response to stimulus. We observed CNVR2181 with four genes showing relatively high value (*V*_*i*_ = 7.71), CNVR190 (*V*_*i*_ = 5.14) and CNVR2618 (*V*_*i*_ = 6.63) overlapped with genes *KPNA6* and *ATP5H*, while CNVR507 overlapped with two genes (*ESPNL* and *SCLY*) (Table 2).

## Discussion

In this study, we performed a genome-wide CNV scan using high density SNP array in Chinese cattle. Recently, many studies regarding CNV discovery had been reported for various cattle populations using aCGH, SNP array and next generation sequencing^25,28,31,39,44–48^. For instance, some previous studies carried out CNV analysis in world-wide cattle including taurine, indicine, and mixed cattle population from African using both BovineSNP50 and BovineHD SNP array^26,44^. In addition, other studies have conducted CNV analyses for local cattle population, including South African Nguni cattle^49^, Brazil Nelore cattle^50,51^, Hanwoo and Japanese Black cattle^52,53^. For Chinese native cattle, only a few studies have been carried out using aCGH and low density SNP array in limited populations^41–43^. Therefore, a comprehensive investigation of CNV and their population genetic properties in diverse Chinese cattle is needed. In the current study, CNV analysis for CDM, WSC, ZTC and NDC was explored for the first time, and the newly discovered CNVs in specific local populations could offer important molecular resources and may further help to elucidate the selection mechanisms of CNV and their genomic adaptation.

In the current study, we detected 13,225 CNV events and 3,356 CNVRs across the cattle genome, of which, 90.33 Mb were newly detected as compared to the cattle HapMap samples^31,44^. In contrast to previous CNV studies in Chinese cattle, we observed 11.38 Mb CNVR was overlapped with previous reports and 136.72 Mb CNVR was newly discovered in present study^41–43^. In addition, we also found 54.76 Mb CNVRs were overlapped as compared to the recent publication in Nelore cattle and 93.34 Mb CNVR were specifically detected in current study^54^. Totally, we detected 4,534 gain and 8,791 loss events across cattle populations in China, and the results shown that deletions are more numerous than duplications which is generally observed in human^55^, mouse^11^, dog^15,56^, and cattle^25,44,45^. This finding may indicate that deletions were more easily to be detected by PennCNV on the BovineHD SNP array^44^. We observed more CNV events were detected in South and Southwest (95.21 and 86.86 per sample) than in North (74.29 per sample) and Northwest (73.76 per sample), and a previous study also detected more CNV events in indicine breed than taurine^26^. This may be because Northern group was more influenced by *B*. *taurus*, and South group was more influenced by *B*. *indicus*, while the cattle in the central and lower areas of the Yellow River and the Huaihe River appear to be a mixture of *B*. *taurus* and *B*. *indicus* types^57,58^. This finding may imply subspecies divergence of genome structures in Chinese cattle. In addition, some of these differences could be related to the fact that SNP markers in BovineHD were designed based on the UMD3.1 reference genome, which may cause bias for CNV calling in indicine-derived cattle populations^45^.

Diversity and selection aspects of CNVs have been extensively explored in world-wide cattle populations using aCGH, SNP array and next generation sequencing, demonstrating that CNVs display breed-specific differences and may be associated with adaptation, health, and production traits^25,31,39,40,46^. To our knowledge, our study is a first attempt to explore the CNV properties using high density SNP array in Chinese cattle across a broad latitudinal range. We suspect CNV are important genomic variants under distinct selection pressures, and CNV can contribute to diverse morphology in cattle sampled from different geographic regions with various environmental factors (e.g., climate, temperature, altitude, rainfall, and food source). Our study explored lineage-differentiated CNVs in Chinese cattle and identified several potential CNV candidates under divergent selection for adaptation in local environmental conditions. CNV genes may have distinct functional roles and be subject to different evolutionary pressures. Interestingly, for shared CNVR with high *V*_*i*_ values across four groups (North, Northwest, Southwest and South), we identified several functionally important genes related to immunity and metabolism, such as *IGLL1*, *CBR1*, and *TKT*. *IGLL1* has been previously detected with copy number changes in cattle genome^40,44^, this gene has been showed differentially expressed in hepatic and mammary tissue in dairy cows^59,60^ and was associated with resistance to gastrointestinal nematodes in Angus cattle^61^. The expression of *CBR1* was found to be associated with oxidative stress in bovine embryos^62^, and this gene had been reported to be associated with growth traits weaning gain in *Bos indicus* ^51^. Previous studies revealed that *TKT* involved in fatty acid synthesis and storage in muscle, which were also correlated with intramuscular fat in both cattle and sheep^63^. The shared CNVs across cattle groups could suggest the parallel selection on CNVs through evolution of cattle genomes, which was also reported in three-spined stickleback^64^. The existence of shared CNVs indicates their potential function may contribute to the parallel adaptive evolution within multiple natural populations in diverse species.

Moreover, our study revealed several group-specific CNV genes, which may play significant roles in diverse morphology and adaptation for local environmental condition. In the North group, we identified several candidate genes related to calcification of cartilage and immune system in MGC and YHC. *TMC6* related to immune system in CNVR2614 (*V*_*i*_ = 4.42) was associated with milk somatic cell score in dairy cattle, which may imply the potential selection for this gene related to the milk quality in cold environmental condition^65^. *COL27A1* within CNVR1360 (*V*_*i*_ = 4.44) may play a crucial role in cartilage calcification and average daily gain^66,67^. Our results indicate these CNV genes could enhance development of bone and muscle, cold tolerance and disease resistance for North group cattle, which live in relatively cold environment in northern China.

For the Northwest group, CDM cattle live on the Qinghai-Tibet plateau in northwestern China, which is an arid environment exhibiting dry, hypoxia, low air pressure. Specifically, we observed several genes *MRVI1*, *ABO*, *GLRB* and *EPHA3* related to nervous system, platelet reactivity, parasite resistance and histoblood group antigens. *MRVI1* overlapped with both CNVR2175 and CNVR2176, and several variants in *MRVI1* had been reported to be associated with platelet count, mean platelet volume and platelet reactivity^68^. In the current study, *ABO*, embedded within CNVR1759 (*V*_*i*_ = 4.90), is related to A and B histoblood group antigens^69^, and copy number changes have been identified within *ABO* in recent studies by Hou *et al*.^26,61^. ABO have also been associated with parasite resistance and susceptibility to gastrointestinal nematodes in Angus cattle^61^. Moreover, a previous study suggested one CNVR containing *ABO*, *SURF6*, *RPL7A* was significantly associated with milk somatic cell score in Holstein cattle^70^. *GLRB*, which was identified as candidate in CDM cattle at BTA17 has been associated with flight speed in beef steers, and *GLRB* protein is a ligand gated ion-channel subunit throughout the central nervous system^71,72^.

In the Southwest group (PWC, LSC and ZTC), we identified several genes *KIT*, *FAT2*, *SLC36A1*, *ZNF331*, *LRFN4* and *RCE1* in four south-specific CNVRs. Notably, *KIT* is a tyrosine kinase receptor, and normal *KIT* signaling is required for development and survival of neural crest-derived melanoblasts^73^. Genetic variation in *KIT* gene has been shown to affect coat coloring pattern in a variety of mammals^74–77^. Rubin *et al*. suggested that white and white spotted pigs are caused by at least two out of the four *KIT* duplications and the porcine *KIT* locus also illustrates the evolution of alleles under strong positive selection^78^. Our results suggested the identified genes may facilitate the cattle living in mountainous areas of Southwestern China.

This study provides a comprehensive investigation of CNV properties in diverse Chinese cattle, and newly identified CNVs contribute to the important genetic resources for the global cattle population. Our results further suggest that lineage-differentiated CNVs may be under divergent selection for adaptation in local environmental conditions. Hybridization array studies may generate both false positive and false negative results, regardless of how the data are analyzed for CNV discovery^79^. Many studies recommend using multiple CNV calling algorithms instead of just one^80^; however, although the net effect of this strategy decreases the false negative rate, it also increases the false positive rate^81^. With advances in next generation sequencing projects, such as the 1000 Human Genomes project^2^ and the 1000 Bull Genomes project^29^, we should be able to better estimate the false positive and false negative rates with better CNV calling standards. Therefore, careful experimental design and rigorous data filtering were required to reveal the impacts of CNVs on both phenotypic variability and diverse selection. Future CNV studies utilizing next-generation sequencing (NGS) and complementary analysis programs will help precisely define the CNV structure and elucidate its function.

## Materials and Methods

### Ethics Statement

All of the animal experiments were approved by the Chinese Academy of Agricultural Sciences (CAAS, Beijing, China). All of the animal procedures were performed in strict accordance with the guidelines proposed by the China Council on Animal Care and the Ministry of Agriculture of People’s Republic of China.

### Sample selection

Blood samples were obtained from 188 individuals representing 8 different cattle breeds across a broad latitudinal range in China. These include Menggu cattle (MGC), Yanhuang cattle (YHC), Caidamu cattle (CDM), Pingwu cattle (PWC), Liangshan cattle (LSC), Zhaotong cattle (ZTC), Wenshan cattle (WSC), and Nandan cattle (NDC). Genomic DNA was extracted from blood samples using the TIANamp Blood DNA Kit (Tiangen Biotech Co. Ltd), and DNA with an A260/280 ratio ranging between 1.8 and 2.0 was subject to further analysis. In this study, we divided 8 breeds into 4 groups based on geographical locations (North group, Northwest group, Southwest group and South group) (Fig. 1). The genotyping platform adopted in this study was Illumina’s Infinium II Multi-Sample Assay. SNP chips were scanned using iScan and analyzed using Illumina’s GenomeStudio 2011. After filtering by the call rate of each given animal (threshold was > 95%), the final data including Log R Ratio (LRR) and B Allele Frequency (BAF) were exported from GenomeStudio software. To avoid the bias of population genetic estimation, genetic relationships between pairwise individuals were estimated using PI-HAT value implemented in PLINK v1.07^82^, unrelated individuals with pairwise PI-HAT < 0.25 were kept for subsequent analyses.

### CNVs detection

In this study, we utilized the PennCNVv1.0.3 software to detect CNV across autosomes in Chinese cattle populations^83^. chrX and chrUn were not considered here due to mapping uncertainty as described previously by Hou *et al*.^26^. The PennCNV algorithm incorporates LRR and BAF, which denote the normalized intensity ratio for each SNP alleles and the frequency of allele B, respectively. The population frequency of B allele (PFB) file was calculated based on the BAF of each marker across populations. The gcmodel file was generated by calculating the GC content of the 1 Mbp genomic region surrounding each marker (500 kb each side). The final CNVs were obtained by filtering the low quality samples with the following thresholds: standard deviation (SD) of LRR as 0.35, BAF drift as 0.01 and waviness factors as 0.05.

### CNVR compilation

CNV regions (CNVRs) were produced by aggregating overlapping CNVs (by at least 1bp) across samples using BEDTools v2.26.0^84^. The CNVRs were classified as “gain”, “loss”, or “both” events. Overlapping “loss” and “gain” CNVRs were merged into single regions to account for “both” events. To facilitate the comparison of CNV pattern among the diverse groups, the CNVRs for each group were generated. The frequency landscape of each group-specific CNVR was visualized using Circos software^85^.

### Function annotation

To elucidate the functional aspect involved with identified CNVs in cattle genome, gene content of cattle CNV regions was assessed using the RefGene track of the UCSC genome browser. Annotation of genes and gene feature analysis (identify the coding sequence and exon of genes presented within CNVs) was performed using the scan_region.pl script from PennCNV v1.0.3 package. PANTHER (Protein ANalysis THrough Evolutionary Relationships) classification system (http://www.pantherdb.org/) was used to explore functional ontology categories as described previously^86^. We tested the hypothesis that the PANTHER molecular function, biological process and pathway terms were under- or over- represented in CNV regions after Bonferroni corrections. Only GO terms with P-value < 0.05 after the Bonferroni correction were considered.

### Signatures of selection

To detect the group-specific CNV events, we proposed a statistic named *V*_*i*_ to estimate the region-specific divergence in CNVR for each group based on unbiased estimates of pairwise *V*_*ST*_, which estimates population differentiation based on average LRR values across all probes falling within a specific CNV region^87^.

For each CNVR, we calculated the statistic ${V}_{i}={\sum }_{j\ne i}\frac{{V}_{st}^{ij}-E[{V}_{st}^{ij}]}{sd[{V}_{st}^{ij}]}$, where $E[{V}_{st}^{ij}]$ and $sd[{V}_{st}^{ij}]$ denote the expected value and standard deviation of *V*_*ST*_ between groups *i* and *j* calculated from the average LRR values across all CNVRs^87^. *V*_*ST*_ is calculated using the following equation: (*V*_*T*_ − *V*_*S*_)/*V*_*T*_, where *V*_*T*_ is the variance in LRR apparent among all unrelated individuals and *V*_*S*_ is the average variance within each group, weighted for sample size. The *V*_*i*_ was inspired by statistic *d*_*i*_ proposed by Akey *et al*.^88^, which is based on the estimation of difference of allele frequency across group. *d*_*i*_ measures the standardized locus-specific deviation using SNP genotype in levels of population structure for a particular group relative to the genome-wide average. Here, we proposed *V*_*i*_ to measure the changes of copy number variant regions (based on average LRR values) for a particular group. Large positive *V*_*i*_ values indicate high levels of group difference of CNV regions relative to the genome-at-large, which are potentially involved with selection. Thus, *V*_*i*_ is particularly well suited for detecting selection of CNV region specific to a particular group.

The Manhattan plot of *V*_*i*_ value for each CNVR across groups were created by the R package “qqman”. To identify the shared and group-specific CNVRs, the Venn diagram was generated based on overlapping of CNVRs with R package “VennDiagram”^89^. Box-plot was generated to display the distribution of average LRR in the identified CNVRs across diverse cattle groups. Unless specified, all statistical analyses were performed using R programming (https://www.R-project.org).

## Electronic supplementary material

Supplementary files

Table S1-S3
