# Supplementary material for: Genome-wide analysis reveals differential selection involved with copy number variation in diverse Chinese Cattle
Source: Sci Rep. 2017 Oct 30;7:14299. doi: 10.1038/s41598-017-14768-0 (PMC5662686; doi:10.1038/s41598-017-14768-0)
Supplement: Supplementary file 1 — Supplementary files [file 41598_2017_14768_MOESM1_ESM.pdf]

Genome-wide analysis reveals differential selection involved with copy number variation in diverse Chinese Cattle

Liu Yang[1,2 †], Lingyang Xu[1,†,*], Bo Zhu[1], Hong Niu[1], Wengang Zhang[1], Jian Miao[1,3], Xinping Shi[1,4], Ming Zhang[2], Yan Chen[1], Lupei Zhang[1], Xue Gao[1], Huijiang Gao[1], Li Li[2], George E. Liu[5], Junya Li[1*]

[1] Laboratory of Molecular Biology and Bovine Breeding, Institute of Animal Sciences, Chinese Academy of Agricultural Sciences, Beijing, 100193, China.

[2] Farm Animal Genetic Resources Exploration and Innovation Key Laboratory of Sichuan Province, Sichuan Agricultural University, Chengdu, 611130, China.

[3] College of Animal Sciences, Fujian Agriculture and Forestry University, Fuzhou, 350002, Fujian, China.

[4] College of Animal Science and Technology, Agricultural University of Hebei, Baoding, 071001, China.

[5] Animal Genomics and Improvement Laboratory, Department of Agriculture-Agricultural Research Services, Maryland, 20705, U.S.

Supplementary methods

Multidimensional scaling (MDS) analysis was conducted using a total of 72,945 SNPs after LD-based pruning (>0.2). Pairwise genetic distances based on IBS values were used to cluster samples across populations with PLINK v1.07 (command line arguments: -mds -plot 4).

Supplementary files

Supplementary File 1

Table S1: The CNVRs on autosomes identified in Chinese cattle including all 8 cattle populations, North group, Northwest group, Southwest group and South group.

Supplementary File 2

Table S2: PANTHER enrichment analysis using a total of 1,400 unique genes which were overlapped with 3,356 CNVR.

Supplementary File 3

Table S3: Genome-wide V_i values for differentiation analysis in four groups (North, Northwest, Southwest and South).

Figure S1

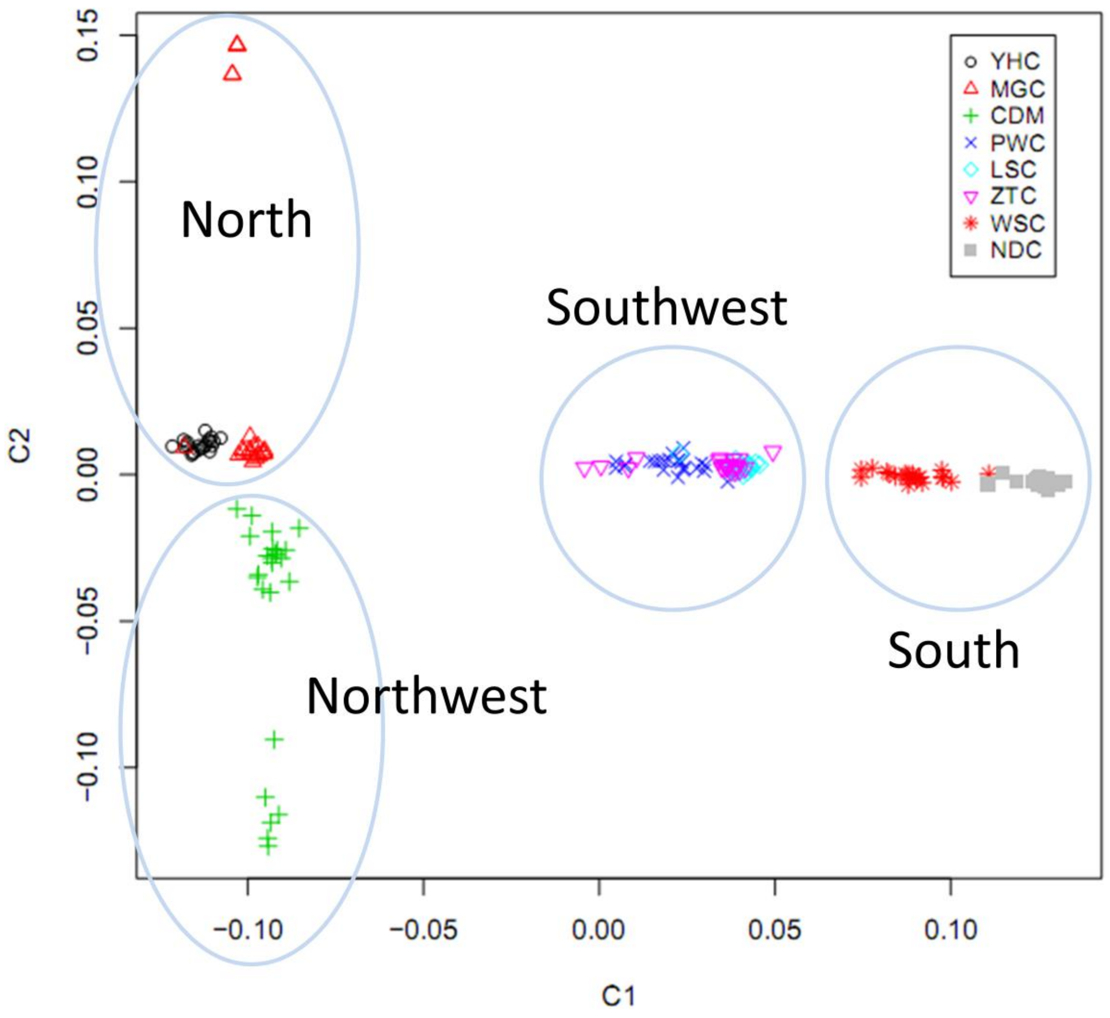

Figure S1 Multidimensional scaling (MDS) analysis results of 166 individuals. Individuals are plotted according to their coordinates on the first two components. MDS analysis was conducted using a total of 72,945 SNPs after LD-based pruning (>0.2).
